# Development of the Functional Connectome Topology in Adolescence: Evidence from Topological Data Analysis

**DOI:** 10.1523/ENEURO.0296-21.2022

**Published:** 2023-02-13

**Authors:** Zeus Gracia-Tabuenca, Juan Carlos Díaz-Patiño, Isaac Arelio-Ríos, Martha Beatriz Moreno-García, Fernando A. Barrios, Sarael Alcauter

**Affiliations:** 1 Instituto de Neurobiología, Universidad Nacional Autónoma de México, Querétaro 76230, México; 2 Centro de Investigación Científica y de Educación Superior de Ensenada, Ensenada 22860, México

**Keywords:** adolescence, functional connectivity, persistence homology, puberty, resting state functional magnetic resonance imaging, topological data analysis

## Abstract

Adolescence is a crucial developmental period in terms of behavior and mental health. Therefore, understanding how the brain develops during this stage is a fundamental challenge for neuroscience. Recent studies have modeled the brain as a network or connectome, mainly applying measures from graph theory, showing a change in its functional organization, such as an increase in its segregation and integration. Topological Data Analysis (TDA) complements such modeling by extracting high-dimensional features across the whole range of connectivity values instead of exploring a fixed set of connections. This study inquires into the developmental trajectories of such properties using a longitudinal sample of typically developing human participants (*N* = 98; 53/45 female/male; 6.7–18.1 years), applying TDA to their functional connectomes. In addition, we explore the effect of puberty on individual developmental trajectories. Results showed that the adolescent brain has a more distributed topology structure compared with random networks but is more densely connected at the local level. Furthermore, developmental effects showed nonlinear trajectories for the topology of the whole brain and fronto-parietal networks, with an inflection point and increasing trajectories after puberty onset. These results add to the insights into the development of the functional organization of the adolescent brain.

## Significance Statement

Topological Data Analysis (TDA) may be used to explore the topology of the brain along the whole range of connectivity values instead of selecting only a fixed set of connectivity thresholds. Here, we explored some properties of the topology of the brain’s functional connectome and how they develop in adolescence. We show that developmental trajectories are nonlinear and better adjusted by puberty status than chronological age, with an inflection point around the onset of puberty. In particular, the results show that the topology of the fronto-parietal network is the one that drives the functional connectome changes in the adolescent period.

## Introduction

Adolescence is a critical development period that substantially impacts the body and behavior. Notably, the brain undergoes structural and functional changes influenced by pubertal hormones (Vijayakumar et al., 2018; Laube et al., 2020). Moreover, these changes occur along with a consolidation of cognitive and executive performance (Baum et al., 2017; Chai et al., 2017).

These insights have been addressed by modeling the brain as a complex network of interacting nodes at task or rest conditions (Biswal et al., 1995; Smith et al., 2009). In this framework, the functional connectome is described by its system properties in biologically plausible terms, mainly using measures from graph theory (Rubinov and Sporns, 2010). Although graph metrics provide an effective framework to describe brain organization properties, they also have some limitations. The main limitation is that graph theory relies on pairwise connections as the basic unit of the network; in this regard high-order interactions within the network cannot be properly handled (Lord et al., 2016). Another potential limitation is the common step of thresholding, which is to discard a subset of the network connections based on several criteria (e.g., statistically, low values, sign, etc.). There is no consensus about this step, and functional connectomes tend to be unstable at different thresholds (Garrison et al., 2015), although group inferences may dramatically change based on thresholding (Gracia-Tabuenca et al., 2020). Nevertheless, other methods have recently been applied to address high-dimensional data, such as Topological Data Analysis (TDA; Sizemore et al., 2018; Expert et al., 2019). TDA models the connectome as a topological space and characterizes its interaction patterns as geometric features, allowing it to simplify complex structures at different scales (Giusti et al., 2015; Santos et al., 2019; Centeno et al., 2022). In particular, TDA applied to functional connectomes is not affected by the potential biases of connectivity thresholding nor brain segmentation (H. Lee et al., 2012; Gracia-Tabuenca et al., 2020); additionally, TDA is a suitable tool to address longitudinal data since it can extract invariant topological features into longitudinal designs (Fülöp et al., 2020). This methodology has been used for over a decade in neuroimaging (Chung et al. 2009; H. Lee et al., 2011, 2012, 2019; Choi et al., 2014; M.H. Lee et al., 2015; Chung et al., 2019) but is not the standard procedure in neuroimaging. Furthermore, it explores different properties to those explored with graph theory or other network approaches that typically explore the fully connected network [when only one single component is present, i.e., when Betti-0 (B_0_) = 1].

In terms of the functional organization of the brain, previous cross-sectional studies have shown that the adolescent period is characterized by an increase in modularity and specialization (Fair et al., 2009; Satterthwaite et al., 2013a; Gu et al., 2015), with prominent effects in frontal and parietal systems, along with executive performance (Marek et al., 2015; Gracia-Tabuenca et al., 2021). However, as far as we are concerned, TDA in human connectomes has mainly been applied to neuropsychiatric disorders (H. Lee et al., 2012, 2017; Gracia-Tabuenca et al., 2020; Li et al., 2021) but not to characterize the typical development. There is still a huge degree of incertitude in this field because of the great variability between samples, sexes, and cultures (Sawyer et al., 2018), with special emphasis on the fact that some individuals have faster or slower pubertal development even when they have the same chronological age (Blakemore et al., 2010; Vijayakumar et al., 2018). In this regard, longitudinal trajectories and pubertal markers are highly valuable in describing adolescent development.

Therefore, this study focuses on how the topology structure of the neurotypical functional connectome changes during the adolescent period. In order to achieve that, TDA features were extracted from the functional brain connectivity of a longitudinal sample of typically developing subjects, and (non)linear trends were tested from chronological age and pubertal status.

## Materials and Methods

### Sample

A general invitation was sent to local schools describing the study protocols and the inclusion/exclusion criteria. Inclusion criteria consisted of a full-term gestation (≥37 weeks). Exclusion criteria included academic year repetition and any neurologic or psychiatric disorder identified with the MINI semi-structured interview. Signed informed consent for parents and verbal assent for minors was required. The study protocols followed the ethical principles of the Declaration of Helsinki and were approved by the Institutional Ethics Board.

**Figure 1.**
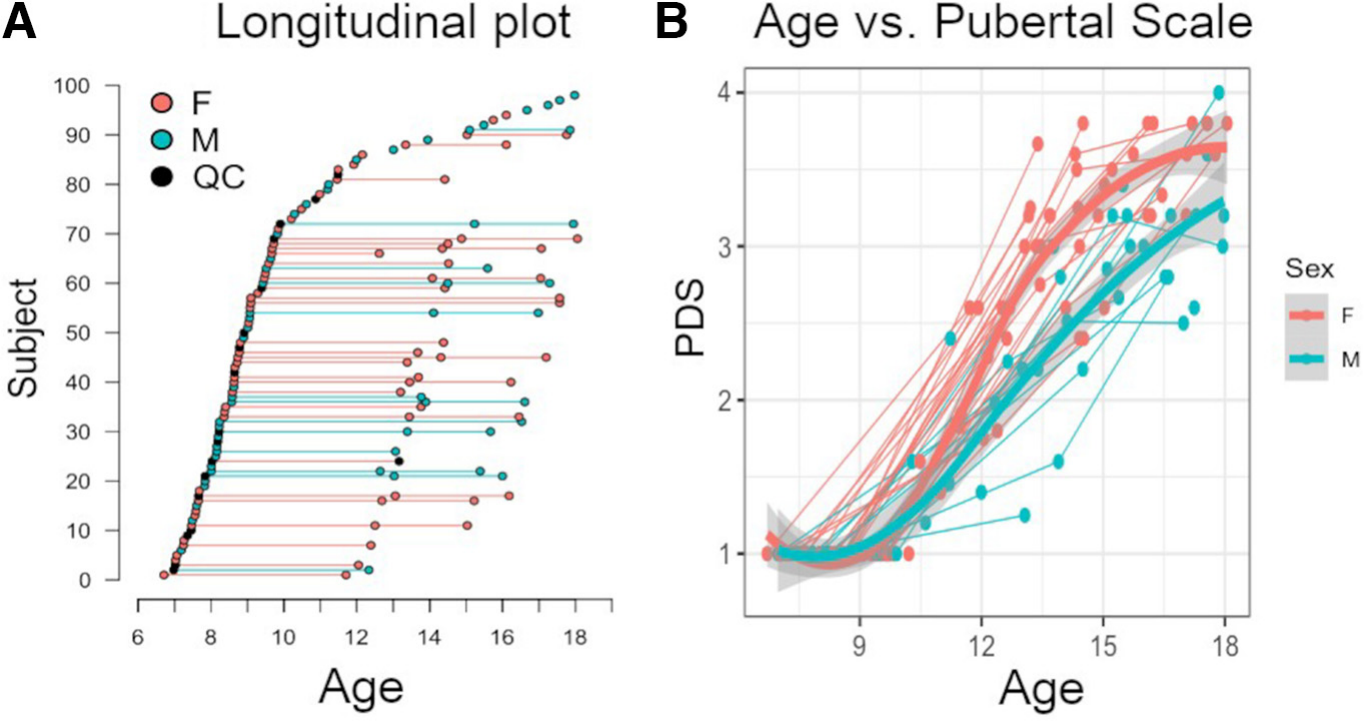
***A***, Longitudinal plot: each dot represents a subject at the age of assessment. Lines represent longitudinal assessments of a subject. ***B***, Pubertal Development Scale scores (PDS) along age. Thin lines represent individual trajectories; thick lines represent locally estimated scatterplot smoothing (LOESS) curves per sex group (with 95% confidence-interval shadow) after controlling for intraindividual trends. F, female; M, male; QC, session with excessive motion artifact. Figures were modified from Gracia-Tabuenca et al. (2021) under CC BY 4.0 license (https://creativecommons.org/licenses/by/4.0/).

The sample comprised 98 typically developing participants (53 females, 45 males; age range: 6.7–18.1 years old). Of those, 41 returned for a second session, and 16 for a third. Initially, the study was designed as a cross-sectional study. However, to include intraindividual development in the growth charts, some participants agreed to participate in one or two follow-up sessions. As a result, follow-ups occurred after five years and the second after two years, respectively (Fig. 1).

### Pubertal status assessment

Participants fulfilled the Pubertal Development Scale (PDS; Petersen et al., 1988). PDS averages the response of five self-reported questions about growth spurt in height, pubic hair, and skin change for both sexes; plus breast growth and menarche for females and facial hair growth and voice change for males. Responses are absence (1), first signs (2), evident (3), and finished (4) pubertal spurt. Those participants under 10 years old were set to PDS level 1, following similar values in previous studies (Hibberd et al., 2015; van Duijvenvoorde et al., 2019). In addition, 8 missing values (four females) were estimated via generalized additive mixed model (GAMM) with age-sex interaction locally estimated scatterplot smoothing (LOESS) curves (according to Gracia-Tabuenca et al., 2021).

### Imaging

Participants underwent an magnetic resonance imaging (MRI) protocol for each session, including a whole-brain functional MRI (fMRI) sequence plus high-resolution T1-weighted images for anatomic reference. After five “dummy” volumes for scan stabilization, a total of 150 fMRI volumes were obtained using a gradient recalled T2* echoplanar imaging sequence (TR/TE = 2000/40 ms, voxel size 4 × 4 × 4 mm^3^). Participants were instructed to lay down, close their eyes, and not fall asleep. In order to ease participants to remain awake, the fMRI scan was applied at the beginning of the MRI session and always in the morning. T1 images were obtained using a 3D spoiled gradient recalled (SPGR) acquisition (TR/TE = 8.1/3.2 ms, flip angle = 12.0, voxel size 1 × 1 × 1 mm^3^). All brain imaging was acquired with a 3T MR GE750 Discovery scanner (General Electric) using an 8-channel-array head coil. However, 20 sessions were acquired with a 32-channel coil; thus, a covariate was included in the subsequent analyses.

### Preprocessing

Structural T1 volumes were denoised with nonlocal means (Manjón et al., 2010) and N4 bias field correction (Tustison et al., 2010). fMRI datasets were preprocessed using FSL v.5.0.6 (Jenkinson et al., 2012; RRID:SCR_002823). Preprocessing steps included slice timing, head motion correction, brain extraction, intensity normalization, confound regression, spatial normalization, and 0.01- to 0.08-Hz bandpass filtering.

Considering that the pediatric population tends to move more inside the scanner (Satterthwaite et al., 2012), we implemented a strident strategy of confounding variables regression (Satterthwaite et al., 2013b). A total of 36 parameters were regressed out from the fMRI time series, including the six head-motion estimated parameters plus the average time series of the global signal, white matter, and cerebrospinal fluid. The derivatives of these nine variables were also added, and the quadratic terms of those eighteen. Additionally, the volumes with a framewise displacement (FD-RMS; Jenkinson et al., 2002) >0.25 mm (“spikes”) were included as confounds as well. This approach overpowers other widely used motion-mitigation methods (Ciric et al., 2017; Parkes et al., 2018; Taymourtash et al., 2019; Graff et al., 2022). Eighteen sessions with <4 min without spike-volumes were discarded (Satterthwaite et al., 2013b; Parkes et al., 2018); therefore, the final sample consisted of 89 participants (39 male, age range: 6.7–18.1 years old), of whom 37 and 11 had two and three longitudinal sessions, respectively.

In addition, fMRI datasets were co-registered to their T1 volume with six degrees of freedom and then warped twice using nonlinear SyN transformation (Avants et al., 2008; RRID:SCR_004757) to a pediatric template (NIHPD4.5–18.5; Fonov et al., 2011) and then to the MNI-152 standard template.

### Functional connectomes

Brain networks were defined based on 264 regions of interest (ROIs) as nodes (Power et al., 2011). Pairwise edges were calculated through Pearson’s correlation between the average fMRI preprocessed signal of every pair of ROIs.

These ROIs consist of 5-mm radius spheres with high consistency in task and rest tested in large fMRI databases (Power et al., 2011). Moreover, this set of ROIs can be grouped in thirteen functional networks. This segmentation has been applied in numerous pediatric studies (Satterthwaite et al., 2013a; Gu et al., 2015; Marek et al., 2015; Chai et al., 2017; Ciric et al., 2017; Gracia-Tabuenca et al., 2020, 2021).

### Topological data analysis

**Figure 2.**
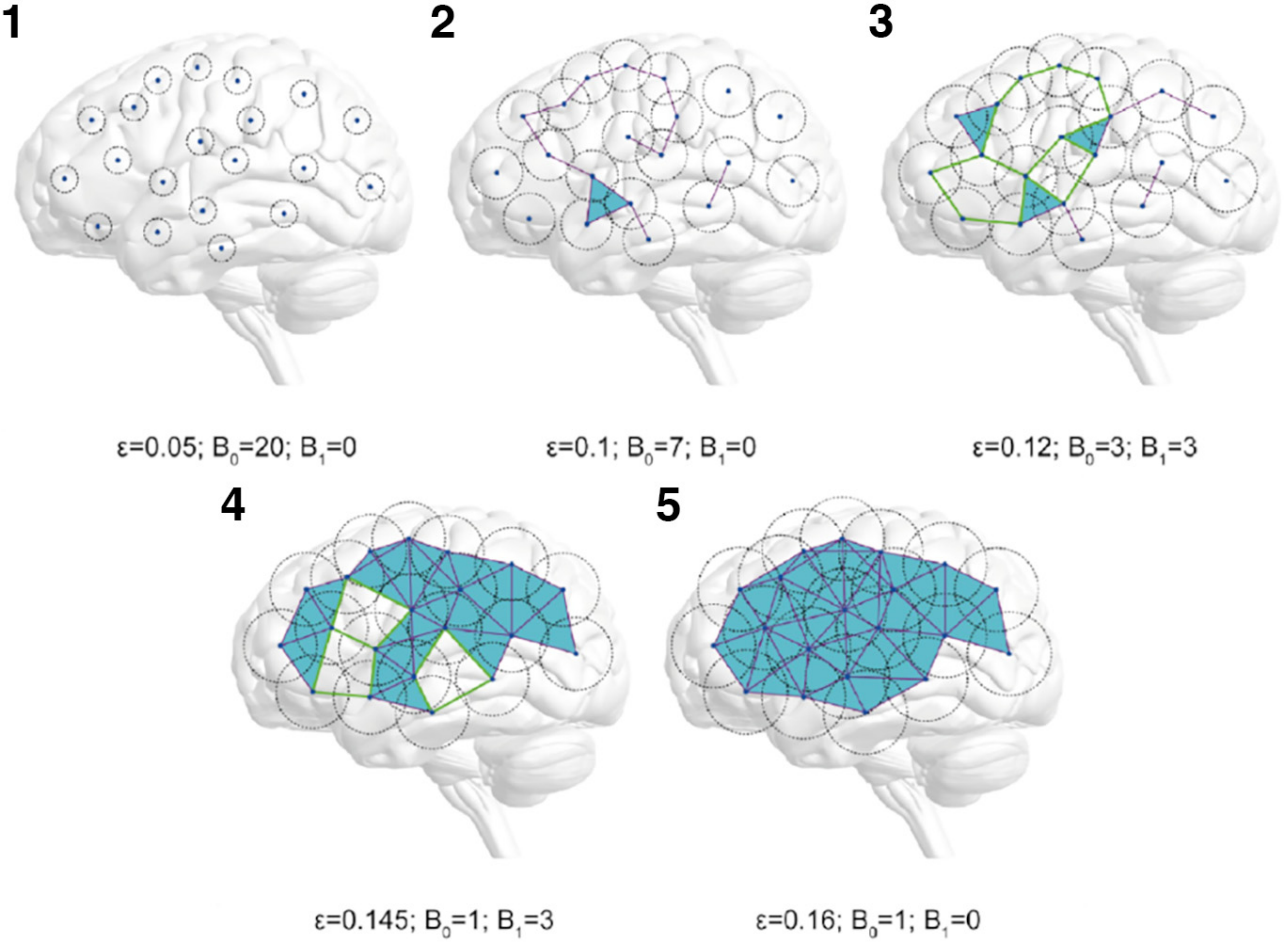
Betti-0 (B_0_) and Betti-1 (B_1_) for different filtration values. Illustrative example with 20 nodes, five filtration values ε, represented as the circle diameter and their corresponding B_0_ and B_1_ values. At ε = 0, the number of components (B_0_) equals the number of nodes, and there are no holes. As the filtration value increases, the number of components (B_0_) decreases until they form a single component containing all the nodes. Meanwhile, the number of holes (B_1_) starts at 0, increases, and finally goes back to 0 again. A hole of dimension 1 is a cycle with four or more edges (highlighted with green edges); the simplices of dimension >1 are colored in light blue for each filtration value ε.

The functional connectome can be modeled as a topological space using the Rips complex, defined as Rips(*F*, *ε*). *F* stands for the set of nodes (same as the connectome nodes), and *ε* stands for the filtration value that indicates if a pair of nodes of *F* are connected (those with a distance lower than *ε*, see below the definition of distance). Thus, the set of connected nodes of the Rips complex varies as a function of *ε*. Additionally, topological features can be extracted from the Rips complexes, the so-called Betti numbers. Specifically, Betti numbers of order zero or Betti-0 (B_0_) accounts for the number of components (i.e., the number of groups of connected nodes and isolated nodes), Betti-1 (B_1_) accounts for the number of 1-dimensional holes which correspond to cycles in the network (Fig. 2), and so on (for an extensive review on TDA, we suggest Sizemore et al., 2019; Edelsbrunner and Harer, 2010).

**Figure 3.**
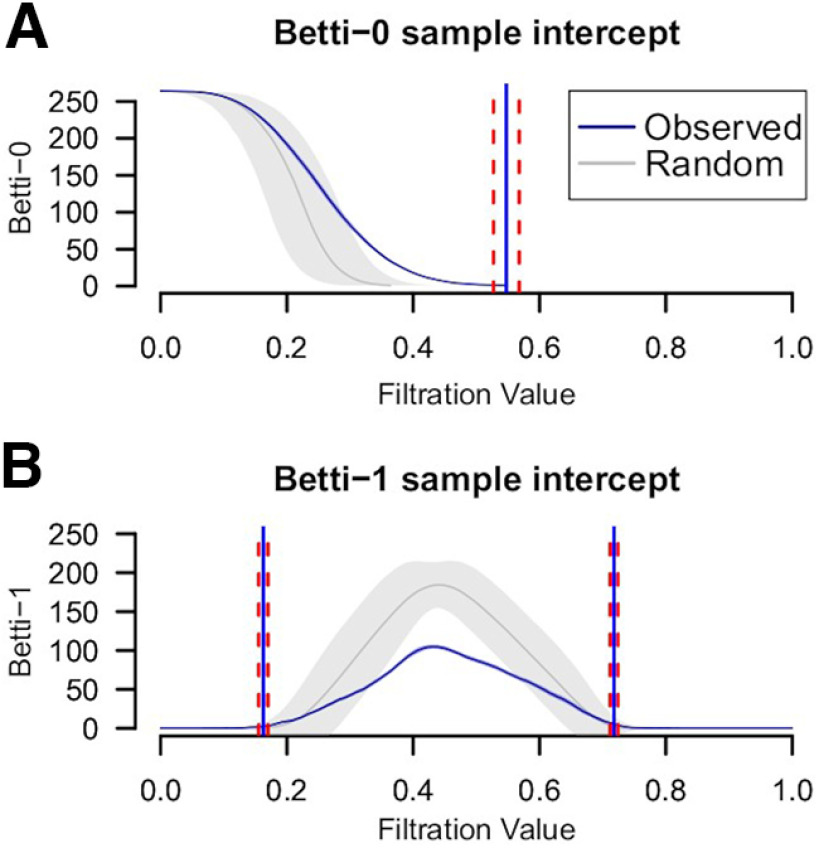
Sample intercepts for the B_0_ (***A***) and B_1_ (***B***) curves are in blue. Average of 1000 bootstrapped connectomes with random edge-rewiring curves in gray. Every average with 95% confidence interval. The blue bars are centered at the sample intercept filtration for starting/ending values with 95% confidence interval in dashed red. Extended Data Figure 3-1 also shows that the persistence of topological holes (B_1_) shown in ***B*** is directly related to the number of holes.

In this study, we focused exclusively on B_0_ and B_1_. When *ε* = 0, B_0_ equals the number of nodes. As *ε* increases, B_0_ decreases and eventually will reach a single component where every node of *F* is connected (Fig. 2). In contrast, at low values of *ε*, there are no holes in the connected pattern of the topological space because there are not enough connections to build them. Similarly, at high values of *ε*, the holes are “filled” because all pairwise connections within the component are accomplished. These holes represent serially distributed connections of nodes without shortcuts, while a filled hole means that those nodes are densely connected between them (Sizemore et al., 2018). Therefore, the greater amount of holes (i.e., B_1_) is reached at intermediate values of *ε*. Both processes can be characterized as Betti curves as a function of *ε* (Fig. 3).

10.1523/ENEURO.0296-21.2022.f3-1 Extended Data Figure 3-1 Scatter-plot of Betti-1 area under the curve (B_1_-AUC) against the number (#) of topological holes, the red line corresponds to the fitted line with its corresponding 95% confidence interval in gray. Intercept at 60.16 and slope of 14.7, *R*^2^ = 0.89 (*p* < 2.2e-16). Intrasubject random-effects were regressed-out from both variables. Download Figure 3-1, TIF file.

The distance between nodes was set as one minus their corresponding Pearson’s correlation (i.e., their functional connectivity edge), following H. Lee et al. (2012): d(ξ, x_j_) = 1 − *r*(ξ, x_j_), *r* being the Pearson’s correlation between nodes ξ and x_j_. B_0_ and B_1_ curves were computed using the TDA R-package (Fasy et al., 2014), and were summarized employing the area under the curve (AUC). This metric, also known as a Persistence Indicator Function and Total Persistence, is a norm associated with the persistence diagrams (Rieck et al., 2020). In addition, stability properties have been proved (Cohen-Steiner et al., 2010), and; it also has been used for the classification of subjects in fMRI datasets (Caputi et al., 2021). The AUC accounts for the overall process of the Betti numbers along all possible values of *ε*. Low scores of B_0_-AUC can be interpreted as a fast transition to the single component. In contrast, higher scores imply a more distributed configuration of the nodes that impedes a rapid transition to a single component. Meanwhile, low scores of B_1_-AUC mean that nodes rapidly bind to one another, fulfilling the topological holes, and higher scores imply an increase in the persistence of such holes, but also in the number of holes within the network (i.e., a less densely connected network; see Extended Data Fig. 3-1).

Furthermore, to discard that the observed results can be obtained by chance, a null distribution of Betti curves was generated by bootstrapping 1000 connectomes extracted from the original sample whose edges were randomly rewired (Giusti et al., 2015; Gracia-Tabuenca et al., 2020).

### Developmental trajectories

Developmental effects were tested using linear mixed-effects (LME) and nonlinear generalized additive mixed models (GAMM). We opted for GAMM because it allows for testing several nonlinear trends simultaneously and can address nonparametric regression estimators (Lin et al., 2004). Additionally, given that PDS is an ordinal and not continuous variable, GAMM is a suitable modeling approach. Eight models were applied: two LME for age and age-sex interaction, six GAMM fitting smooth splines for age and age-by-sex, PDS and PDS-by-sex, and Age-PDS interaction and Age-PDS interaction-by-sex. The longitudinal dimensions of the sample were modeled by intraindividual intercepts, which accounted for the random-effects of the models, and were estimated via maximum likelihood. Additionally, every model included the average head motion (FD-RMS) and coil as confounds. Models were implemented using R libraries: LME via *lme4* (Bates et al., 2007; RRID:SCR_015654), GAMM via *gamm4* (Wood et al., 2017). The selection of the best model was set by the lowest Akaike Information Criterion (AIC; Akaike, 1974). The AIC evals a model by the trade-off between its complexity and its goodness of fit. That is, the subtraction between the number of parameters (*k*) and the log-likelihood function (*lnL*) by a factor of two (i.e., AIC = 2*k* − 2*lnL*). Thus, based on information theory, the best model is the one with the minimum AIC value. Model assumptions were evaluated with Shapiro–Wilk normality tests on the residuals and random-effects (Verbeke and Lesaffre, 1996). Developmental terms within the model with lower AIC were tested via *F* tests (Wood, 2013).

In addition, the model with lower AIC at the whole-brain level was applied for the thirteen functional networks of the Power et al. (2011) segmentation, where their corresponding significance was corrected for multiple testing using a false discovery rate (FDR) q < 0.05 (Benjamini and Hochberg, 1995).

### Code accessibility

All preprocessed data and the code described in this study are freely available online at https://github.com/BrainMapINB/Pubertal_TDA. Also, the code is available as Extended Data 1. Present results were computed with an Intel Core i7-4790 CPU @ 3.60 GHz × 8 with Ubuntu 18.04.3 LTS 64-bit.

10.1523/ENEURO.0296-21.2022.ed1 Extended Data 1 Code and materials. Download Extended Data 1 ZIP file

## Results

The sample intercept B_0_ curve, the representative curve for the whole sample, showed an inverse sigmoid pattern with a slower transition to the single component compared with the permuted data (Fig. 3*A*), which means that the brain topology is more complex compared with a random network. On the other hand, the B_1_ curve shows a bell shape with a maximum of 104.69 “holes” at 0.432 filtration value, while the permuted data shows a maximum of 183.92 at *ε* = 0.438 (Fig. 3*B*). This implies that the brain network has a more densely connected pattern, compared with random networks, and therefore less number of topological holes.

In addition, the average filtration value where B_0_ curves get to the value of 1 is 0.5474, with a 95% confidence interval of [0.527, 0.5677] (Fig. 3*A*). The average filtration value where B_1_ curves begin to increase above 0 is 0.1627, with a 95% confidence interval of [0.1554, 0.1699]. Similarly, the average filtration value where B1 curves return to 0 is 0.718, with a 95% confidence interval of [0.7118, 0.7242] (Fig. 3*B*). This information means that both the B_0_ and the B_1_ curves start and end around the same filtration values. That is, two Betti curves with similar areas will not have extremal differences in their curve profiles.

**Figure 4.**
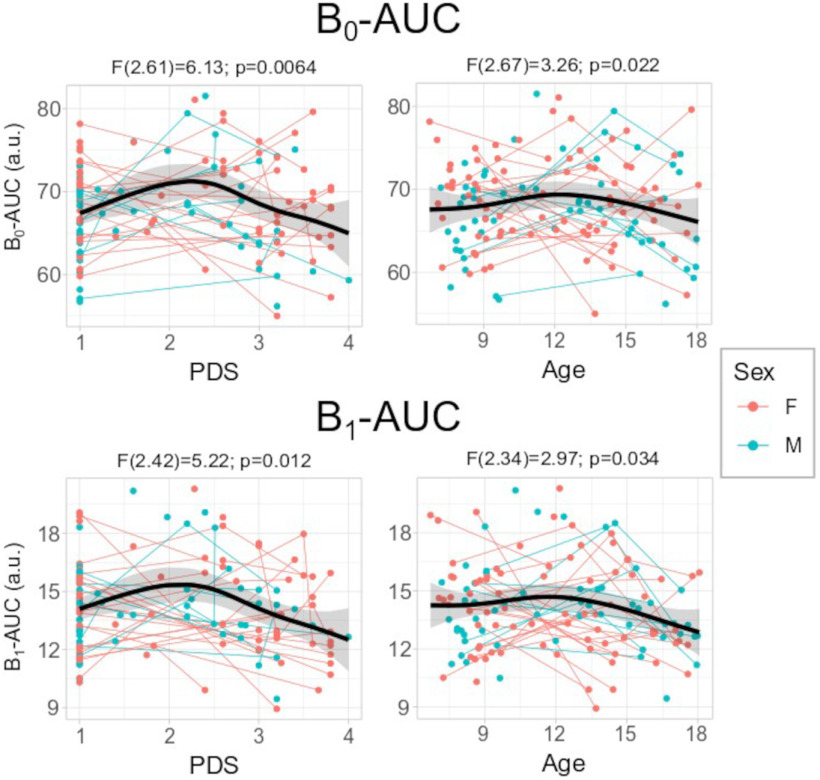
Scatter-plots of the GAMM PDS (left) and age (right) models for the B_0_ (top) and B_1_ (bottom) area under de curve (AUC) in relation to the pubertal scale (PDS) or age (in years). Thin lines represent individual trajectories; thick black lines represent the sample curve (with 95% confidence-interval shadow). Smooth spline *F* statistics are depicted. GAMM-age adjustments have higher AIC than GAM-PDS, but are depicted as a reference. a.u., arbitrary units. The relationship between the PDS or Age and the TDA features residuals after regressing out average motion, head coil, and intraindividual slopes covariates is included in Figure Extended Data Figure 4-1. F, females; M, males.

Regarding model selection for the developmental effects, the GAMM for the PDS showed the lowest AIC for B_0_-AUC (858.3) and B_1_-AUC (625.6; Table 1). Additionally, the normality assumption was rejected (based on the nominal α = 0.05) only in the residuals of the linear mixed-models of the B_1_-AUC, but the assumption remains for all of the nonlinear models as well as all B_0_-AUC models tested. The B_0_-AUC and B_1_-AUC trends along the PDS show an initial increase from level 1 to 2, followed by a soft decline after that (Fig. 4). Both effects were significant (B_0_-AUC: *F* = 6.13, EDF = 2.61, *p* = 0.0064; B_1_-AUC: *F* = 5.22, EDF = 2.42, *p* = 0.012).

**Table 1:** Akaike Information Criterion (AIC), Shapiro–Wilk test (SW) for the residuals (e) and random-effects (RE) for Betti-0 (B_0_) and Betti-1 (B_1_) areas under the curve (AUC) at every developmental model

	B_0_-AUC			B_1_-AUC		
	AIC	e-SW(p)	RE-SW(p)	AIC	e-SW(p)	RE-SW(p)
LME-Age	863	0.993 (0.71)	0.984 (0.36)	627.6	0.98 (0.04)	0.974 (0.07)
LME-Age.Sex	864.9	0.994 (0.82)	0.984 (0.35)	631.3	0.98 (0.04)	0.974 (0.07)
GAMM-Age	861.4	0.994 (0.79)	0.987 (0.53)	627.7	0.986 (0.18)	0.981 (0.22)
GAMM-Age.Sex	862.9	0.994 (0.82)	0.986 (0.49)	630.9	0.987 (0.21)	0.981 (0.22)
GAMM-PDS	858.3	0.992 (0.65)	0.983 (0.31)	625.6	0.989 (0.36)	0.988 (0.63)
GAMM-PDS.Sex	859.5	0.991 (0.57)	0.979 (0.15)	633.1	0.985 (0.14)	0.984 (0.37)
GAMM-AgePDS	859.4	0.994 (0.82)	0.983 (0.3)	625.9	0.991 (0.54)	0.987 (0.53)
GAMM-AgePDS.Sex	862.5	0.993 (0.74)	0.983 (0.3)	631.7	0.991 (0.57)	0.979 (0.17)

Developmental models: linear mixed-effects models for age (LME-Age) and age-sex interaction (LME-Age.Sex), generalized additive mixed models with smooth splines for age (GAMM-Age) and age-by-sex (GAMM-Age.Sex), PDS (GAMM-PDS) and PDS-by-sex (GAMM-PDS.Sex), and age-PDS interaction (GAMM-AgePDS) and age-PDS interaction-by-sex (GAMM-AgePDS.Sex). Akaike Information Criterion (AIC) and Shapiro–Wilk tests (SW) for the residuals (e) and random-effects (RE) of the Betti-0 (B0) and Betti-1 (B1) areas under the curve (AUC) for every developmental model.

**Figure 5.**
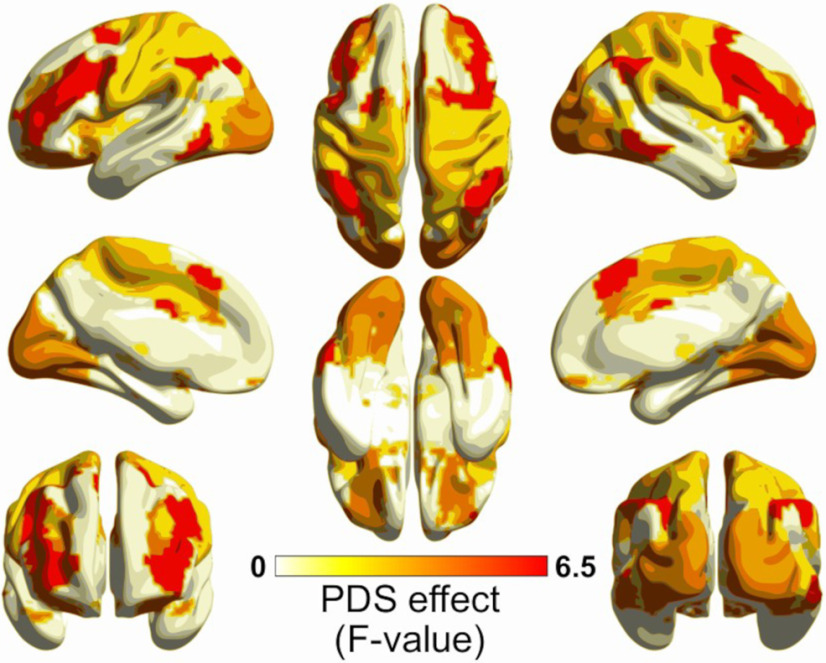
Brain maps of the smooth spline’s *F* value of the Pubertal Development Scale (PDS) GAMM term for B_0_-AUC at the functional networks level. Mapping was based on ROI corresponding to the consensus area according to Power et al. (2011), using BrainNet Viewer (Xia et al., 2013; RRID:SCR_009446).

**Figure 6.**
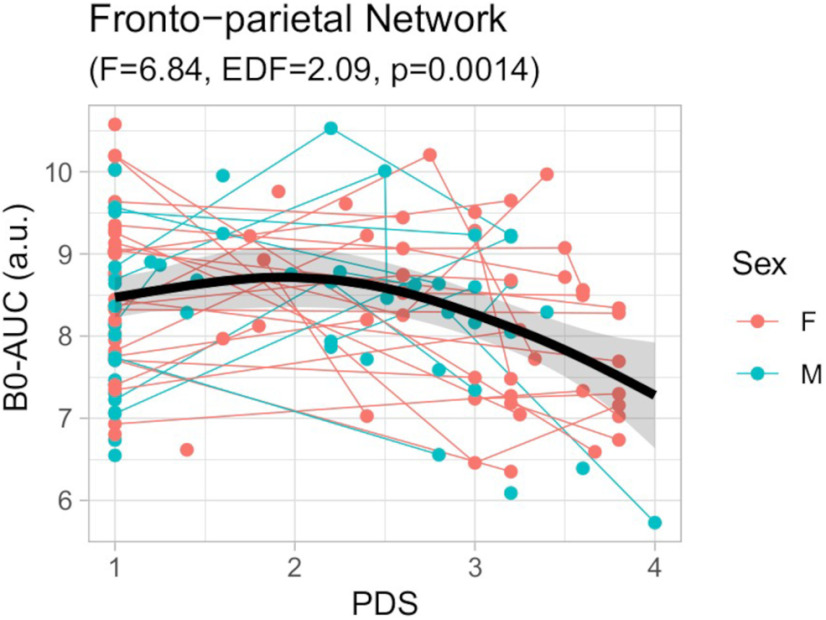
Scatter-plot of the GAMM model for the B_0_-AUC of the fronto-parietal network (FPN) in relation to the pubertal scale (PDS). Thin lines represent individual trajectories; thick black lines represent the sample’s smooth spline curve (with 95% confidence-interval shadow). Smooth spline *F* statistics are depicted. a.u., arbitrary units. The relationship between the PDS and the B_0_-AUC residuals after regressing out average motion, head coil, and intraindividual slopes covariates is included in Extended Data Figure 6-1. EDF, effective degrees of freedom.

Concerning the developmental effects at the functional network level, the PDS term showed strong effects in the fronto-parietal (FPN) and moderate effects in the auditory (AUD), sensorimotor-hand (SMH), and subcortical (SUB) networks for the B_0_-AUC (Fig. 5). Only the FPN had a significant effect after FDR correction (*F* = 6.84; EDF = 2.09; *p* = 0.001), which shows a nonlinear trend similar to the whole brain network (Fig. 6). No effects (even uncorrected) were found for the B_1_-AUC.

10.1523/ENEURO.0296-21.2022.f4-1 Extended Data Figure 4-1 Scatter-plots of the GAMM PDS (left) and age (right) models for the B_0_ (top) and B_1_ (bottom) area under de curve (AUC) residuals (e) in relation to the pubertal scale (PDS) or age (in years). Average motion, head coil, and intraindividual slopes were regress-out from the dependent variable. Thin lines represent individual trajectories; thick black lines represent the sample curve (with 95% confidence-interval shadow). Smooth spline *F* statistics are depicted. GAMM-age adjustments have higher AIC than GAM-PDS, but are depicted as a reference. Download Figure 4-1, TIF file.

10.1523/ENEURO.0296-21.2022.f6-1 Extended Data Figure 6-1 Scatter-plot of the GAMM model for the B_0_-AUC residuals (e), after regressing out in-scanner motion and head-coil, of the fronto-parietal network (FPN) in relation to the pubertal scale (PDS). Thin lines represent individual trajectories; thick black lines represent the sample’s smooth spline curve (with 95% confidence-interval shadow). Smooth spline F statistics are depicted. Download Figure 6-1, TIF file.

**Table 2:** Model performance at the RSN, effective degrees of freedom (EDF), *F* and *p* values for the development component

	B_0_-AUC			B_1_-AUC		
	EDF	*F*	*p*	EDF	*F*	*p*
AUD	1	0.47	0.493	1.93	1.82	0.156
CBL	1	0.04	0.838	1	1.16	0.283
CON	1.91	2.38	0.067	1	0.44	0.506
DMN	1	0.37	0.543	1	0.02	0.896
DAN	1.78	1.57	0.203	1	0.01	0.904
FPN	2.09	6.84	0.001*	1	3.21	0.076
MEM	1	0.07	0.794	1	2.03	0.157
SAL	1	4.09	0.045	1	0.82	0.368
SMH	1	2.74	0.1	1	0.04	0.839
SMM	1	1.83	0.178	1	0.26	0.611
SUB	1	1.98	0.162	1	0.24	0.628
VAN	1	0.16	0.691	1	1.12	0.291
VIS	1	3.81	0.053	1	4.01	0.047

Auditory (AUD), cerebellar (CBL), cingulo-opercular (CON), default mode (DMN), dorsal attention (DAN), fronto-parietal (FPN), memory-retrieval (MEM), salience (Sal), sensomotor-hand (SMH), sensomotor-mouth (SMM), subcortical (Sub), ventral attention (VAN), and visual (VIS) networks.

* Significant after FDR-corrected at q < 0.05.

## Discussion

In this study, we have applied Topological Data Analysis (TDA) on the functional connectomes of a longitudinal sample of typically developing children and adolescents. TDA features show a more distributed connectivity structure compared with random networks. However, when assessing the connectivity structure within the connected nodes, the brain connectomes exhibit a more densely connected pattern than the random networks. Furthermore, this topology develops nonlinearly through adolescence, better adjusted to pubertal status than chronological age. This nonlinear effect exhibits a stronger connectivity of the whole-network and within the fronto-parietal network just after the onset of the pubertal signs.

Regarding the average Betti curves, the sample intercept for the B_0_ curve showed an inverse sigmoid pattern that replicates previous findings in functional connectivity fMRI (Liang and Wang, 2017; Gracia-Tabuenca et al., 2020; Li et al., 2021) and PET (H. Lee et al., 2012) studies. Furthermore, the average B_0_ curve of the randomized data reached the single component faster than the observed data, evincing a less distributed network. This random pattern was also replicated in another pediatric sample showing the same faster transition to a single component (Gracia-Tabuenca et al., 2020). Concerning the B_1_, both the sample intercept and randomized curves exhibit a bell-shaped curve with an approximately similar filtration value at their maxima but at a lower area for the observed data. This information evinces a connectivity topology structure of a lower number of holes or more densely connected at the local level in the real data compared with the random networks. Thus, B_0_ tells how fast the whole-network goes from isolated to all-connected nodes, while B_1_ reflects how densely connected those elements are already connected. It is noticeable that, on average, B_0_ tends to reach the single component at filtration values of *ε* = 0.5, but at that point, B_1_ curves display their peak number of “holes.” This information implies that these TDA features not only reflect different levels of connectivity structure but also occur at different connectivity strengths.

Regarding the developmental effects of the TDA features, several models were tested to address the area under the B_0_ and B_1_ curves. Nonlinear additive models show greater goodness of fit compared with linear ones, even controlling for the extra number of parameters. Specifically, when considering the pubertal status (assessed by the PDS) without its sex interaction is the model that better fits the AUC for both B_0_ and B_1_. Hence, the development of the functional connectome topology better adjusts pubertal status than chronological age. This is a relevant finding considering that the pubertal status takes into account noncontinuous changes as well as more subtle sex effects than a nonlinear age-sex interaction. No previous studies have addressed the adolescent connectome via TDA, but recent studies have shown a better adjustment with the pubertal status for the developmental trends of the functional connectome (based on graph theory; Gracia-Tabuenca et al., 2021) or the frontostriatal functional connectivity (van Duijvenvoorde et al., 2019). Previous studies in animal models have shown brain plasticity associated with puberty-related hormonal changes (Sisk and Foster, 2004). Neuroimaging studies controlling for the age effects have also revealed structural changes associated with the puberty stage in humans, mainly showing decreased gray matter density but increased white matter density in later stages (Peper et al., 2009; Perrin et al., 2009; Bramen et al., 2011; Herting et al., 2012). This work contributes to the emerging evidence that puberty onset dramatically influences the development of functional brain connectivity (van Duijvenvoorde et al., 2019; Gracia-Tabuenca et al., 2021).

Concerning whole-brain inferences, the AUC for both B_0_ and B_1_ showed an initial increase from PDS level 1 to 2, but decreased afterward. In contrast, when focusing on the chronological age, the turning point is ∼12 years old, but showing smoother trends compared with PDS. This means a faster transition to the single component for the B_0_ while a lower rate of geometric holes for the B_1_ at the end of adolescence when considering PDS or age. But only B_0_-AUC effects were significant, which evinces that those changes were more prominent at lower filtration values, i.e., edges with higher functional connectivity (see Materials and Methods, Topological data analysis). Previous work on brain functional organization during this period has shown increases along age in the within-network connectivity (Fair et al., 2009; Satterthwaite et al., 2013a; Gu et al., 2015), while others failed to replicate that pattern showing an increase in the between-network functional connectivity instead (Hwang et al., 2013; Marek et al., 2015). In addition, when considering the PDS, it has been shown that functional centrality, segregation, efficiency, and integration increase at the end of adolescence (Gracia-Tabuenca et al., 2021). Such divergent results in neurotypical samples can be explained by the use of different samples and methods, such as selection of regions/networks of interest, thresholding, and/or connectome features. However, the present study uses longitudinal nonlinear modeling with features from the TDA framework, which is resilient thresholding and can extract high-order patterns at the local and global levels at the same time. All these studies demonstrate the change in the configuration of the brain’s functional organization during adolescence.

Likewise, the B_0_-AUC along the PDS effect was stronger in the Fronto-Parietal Network (FPN), showing a similar nonlinear trend as that for the whole brain network (Table 2; Figs. 5 and 6). This demonstrates a faster binding of the FPN nodes at the end of adolescence in terms of functional connectivity. The FPN is a key module of the connectome that is involved in the response to high-demanding tasks (Zanto and Gazzaley, 2013) and is a fundamental system for the consolidation of executive behavior in the adolescent period (Baum et al., 2017; Chai et al., 2017). Other works on functional connectomes have reported an increase in the FPN connectivity along with other attention-related systems in late adolescence (Hwang et al., 2013; Marek et al., 2015). In fact, when applying the graph theory measures to this sample (in a previous study from our group, Gracia-Tabuenca et al., 2021), we found diverse connectome changes in several functional networks, including attention-related but also primary and subcortical, but when using TDA features the central role of the FPN is clearly stated among the other networks.

Some limitations of this work should be taken into account. We used relatively short scans, which may affect the quality of the data; nonetheless, it was considered sufficient at the time of the first acquisition (Van Dijk et al., 2010). Furthermore, we applied a strident quality control of the data discarding those datasets with <80% good quality data in terms of motion artifact. Nonetheless, Topological Data Analysis (TDA) complements other network modeling strategies by extracting high-dimensional features across the whole range of connectivity values, instead of exploring a fixed set of connections.

In conclusion, the present study focused on the characterization of functional connectomes as topological spaces in a longitudinal sample of typically developing children and adolescents. Observed Topological Data Analysis (TDA) features showed a more distributed structure but with denser local connections compared with random networks. However, during adolescence, this effect changes with a nonlinear trend for the whole-brain and the fronto-parietal network, particularly after the onset of the pubertal signs. These results provide evidence of the nonlinear, puberty-dependent developmental trajectories of the topology of the brain network. With the advantage that these properties arise when exploring the whole range of connectivity strengths instead of focusing on a small set of them. Although, as far as we are concerned, this is the first implementation of TDA into neurotypical development, we have shown that this approach can handle complex data in a multi-session design and effectively detect meaningful changes in the adolescent functional connectome. Being adolescence a critical period for the appearance of the first signs of mental health disorders, we expect these trajectories may be of interest for studying both normal and altered development.

## Synthesis

Reviewing Editor: Christie Fowler, University of California, Irvine Decisions are customarily a result of the Reviewing Editor and the peer reviewers coming together and discussing their recommendations until a consensus is reached. When revisions are invited, a fact-based synthesis statement explaining their decision and outlining what is needed to prepare a revision will be listed below. The following reviewer(s) agreed to reveal their identity: NONE.

The study investigates changes in the brain functional connectome that occur during adolescence using algebraic topology, specifically connectivity of simplicial complexes (Betti numbers b0 & b1). The methodological framework is original and may be helpful to derive information about brain networks segregation and integration. Some other aspects are commendable such as the longitudinal sample and the quite high sample size. The conclusions stipulate that as adolescence goes the brain becomes more segregated at the global level and more densely connected at the local level, and that the trends are nonlinear.

The study may make an interesting contribution to the field of human brain mapping, although these finding are not particularly new. However, the conclusions may not be sufficiently supported by the results. Moreover, the study is not strongly motivated (no question, no hypothesis

Specific comments:

1. The article suffers from a lack of insight on what TDA reveals about the longitudinal growth patterns that other tools don’t reveal.

2. Parametric statistical approaches like LME have stringent model assumptions. No model checking details are included.

3. I don’t feel confident that the topological metrics are really about networks segregation and integration, i.e. independent processing in specialized subsystems and global cooperation between different subsystems, respectively. As far as I understand them they likely relate to these definitions, but not sure to which extent. My concern applies particularly to integration, which cannot be considered by applying TDA per network (and accordingly ignoring interaction between networks). This naturally leads to questionable interpretations.

4. The absence of any research question and hypothesis (as noted above) makes the relevance and impact of the study outcomes difficult to evaluate. In particular, it’s hard to judge whether the statistical framework is the most appropriate. The authors should expose clearly the rational behind model selection (especially why nonlinear trajectories have been examined using GAMM models and not more specific model-driven approaches).

5. Several points about LME/GAMM modelling need to be addressed:

(i) the authors discuss the goodness of fit of the different classes of models but terms of most of them are not significant. This approach seems questionable to me.

(ii) a conclusion is that nonlinear models surpass linear ones, and that development effects show nonlinear developmental trajectories. However, results did not demonstrate it for age and age.sex, and only nonlinear models have been implemented for PDS and PDS.sex. I have difficulty to understand from where this conclusion come from.

(iii) the respective impact of age and puberty on network topological characteristics is difficult to established since both variables largely covary (I guess) and have not been tested jointly in the models (e.g. controlling for one while testing the other). Statements as “the connectome topology better adjusts the pubertal status than chronological age” appears overstated to me for now.

6. Overall, data are not described with enough details. The authors should better describe the longitudinal sample, the way the longitudinal dimension of the sample have been modelled (how repeated measurements have been taken into accounts), the relationship between PDS and age, the extent of censored brain data, the output of all models (LRT, intercept, p-values, etc).

7. Outputs from the models are represented as standardized residuals so that it’s tricky to get an idea of the real relationships between AUC-B0/B1 and PBS or age (beyond saying that the trend is nonlinear). It would be important to provide further details on the exact nature of the relationships between AUC-B0/B1 and PBS or age.

8. It would be important to improve Figure 1 and text related to it to make easier the intuition about B0 and B1 in terms of brain functioning.

9. The result section is very short and needs to be revised to present outcomes in a more exhaustive and comprehensive way.

10. The author should discuss in more details what is original in their findings with respect to existing literature. As the discussion stands currently, it seems that their study only confirms to some extent already familiar results.

11. Technical details related to TDA need to be improved, e.g.

- Line 148: “which nodes [of F]” should be “which pairs of nodes”;

- Line 152: “number of components” should be “number of connected components”;

- Line 165: “Blue triangles are not holes as they are surfaces between triads of nodes” is inaccurate in the context of Figure 1 as the two-dimensional complex doesn’t contain tetrahedrons;

- Line 169: “in favor of the connected nodes” should be “as the number of connected components, B0, increases”.
